# Dynamics of a Gel-Based Artificial Tear Film with an Emphasis on Dry Disease Treatment Applications

**DOI:** 10.3390/gels7040215

**Published:** 2021-11-16

**Authors:** Hamza Mehdaoui, Hamid Ait Abderrahmane, Clement de Loubens, Faïçal Nait Bouda, Sofiane Hamani

**Affiliations:** 1 Laboratoire de Mécanique, Matériaux et Energétique, Faculté de Technologie, Université de Bejaia, Bejaia 06000, Algeria; hamzamehdaoui21@gmail.com (H.M.); naitboufa@hotmail.com (F.N.B.); 2 Department of Mechanical Engineering, Khalifa University, Abu Dhabi P.O. Box 127788, United Arab Emirates; 3 Univ. Grenoble Alpes, CNRS, Grenoble INP, LRP, 38000 Grenoble, France; clement.de-loubens@univ-grenoble-alpes.fr; 4 Laboratoire de Recherche en Hydraulique Appliquée et Environnement, Faculté de Technologie, Université de Bejaia, Bejaia 06000, Algeria; hamani.sofiane@gmail.com

**Keywords:** dry eye disease, gel-based artificial tears, Herschel–Bulkley model, tear film breakup, VOF method

## Abstract

This paper discusses the spreading of gel-based ophthalmic formulation on the cornea surface assumed to be flat. We show that gel-based formulations exhibit rheological behaviors that the Herschel–Bulkley model can describe. The continuity and momentum equations are solved numerically using the monofluid formulation and the volume-of-fluid (VOF) method. We investigated the influence of the rheological properties, namely the consistency, the yield stress, and the flow behavior index, on the spreading of a gel-based artificial tear over the cornea surface. We propose optimal values of these properties for efficient gel-based artificial tears.

## 1. Introduction

The tear film spreads across the cornea surface to keep it wet and lubricate and protect eyes from infections, dirt, and dust. The tear film includes lipid, aqueous, and mucus layers [1]. The lipid layer, secreted by the meibomian glands, flows above the aqueous layer to reduce the aqueous layer’s drainage and evaporation at the end of the blinking phase. The aqueous layer, which constitutes most of the tear volume, flows over the precorneal mucus layer. The latter is the deepest layer of the tear film; it allows the aqueous layer to adhere to the cornea.

Tear film instability and tear evaporation can lead to tear film breakup (TBU) and result in a transient spike in saltiness that can cause inflammation of the cornea surface [2,3]. TBU occurs when a tear film thins to the point that the lipid layer touches the cornea surface. Clinically, TBU is associated with the dark spots observed following the instillation of fluorescein dye. TBU can be caused by tear evaporation, Marangoni-driven tangential flow, and dewetting due to a defective corneal surface [4]. TBU and ocular surface inflammation are thought to be the core mechanisms of dry eye disease (DED) associated with sensations of discomfort, visual disturbance, irritation, foreign body sensation, light sensitivity, and watering of the eyes [5,6]. DED can significantly affect a person’s quality of life, such as reading, driving, and computer use [7]. DED is a widespread ophthalmic condition affecting approximately 5–50% of the population globally [2,3]. Nowadays, because of prolonged exposure to the screens of electronic devices, DED affects persons of diverse ages, including children [8,9].

Severe DED can be caused by health conditions such as diabetes, rosacea, Sjögren’s syndrome, rheumatoid arthritis, lupus, and scleroderma, which might require medication adjustment and anti-inflammatory drugs [10]. Artificial tear supplementation is the first-line therapy for mild DED. Artificial tears increase tear volume, minimize eye dehydration, reduce tear osmolarity, and lubricate the ocular surface. In addition, they provide temporary relief for various mild symptoms of DED, such as temporary improvement in eye irritation, tear breakup time, and corneal surface regularity [11]. According to Business Wire (https://www.businesswire.com/news/home/20210709005371/en/Global-Artificial-Tears-Market-Forecast-2020-to-2028---COVID-19-Impact-and-Analysis---ResearchAndMarkets.com, last accessed 9 July 2021), the artificial tears market was valued at USD 2612.91 million in 2020 and is projected to reach USD 3961.63 million by 2028.

Ideal artificial tear formulation should mimic natural tears and have similar physicochemical properties, such as viscosity, surface tension, lubricity, retention time, and adhesion to the ocular surface [12]. Specific artificial tear formulations contain lipids that decrease the surface tension, retard the tear film evaporation, and increase the tear film stability [13]. Tear viscosity is an important parameter that plays a crucial role in the film dynamics of tears. Natural tears exhibit non-Newtonian behavior; they manifest low viscosity at high shear rates to prevent damage to the ocular surface during blinking and exhibit higher viscosity when the eye is fully open to resist drainage and tear film breakup [14].

There are various artificial tears on the market; they differ in their rheological behavior from instillation, through the blink cycle, and back to rest. Water-based artificial tears exhibit Newtonian behavior; their viscosity is relatively low and remains constant during all the phases of blinking. The viscosity of these products ranges from 1.5 to 2 millipascal-second (mPa·s) and approaches even the viscosity of water (1 mPa·s) [15,16]. In addition, water-based artificial tears have a short ocular residence time and provide short, temporary relief from DED symptoms [14].

There are also artificial tears that try to mimic the non-Newtonian behavior of normal tears. These tears are 10 to 100 times more viscous than water-based artificial tears. The viscosity of these solutions decreases with shear stresses during the phases of blinking [15,16]. Some non-Newtonian artificial tears exhibit yield-shear behavior, which means these tears flow only when the shear stress is above the yield shear. The viscosity of these solutions is very high at rest (10^5^ to 10^6^ mPa·s); it decreases by a factor of 1 to 4 when the applied shear is above the yield-shear stress [15,16]. In addition, the viscosity of these solutions drops during the blink but never falls below a certain threshold (around 10 mPa·s, or ten times the viscosity of water).

Artificial tear formulations are often tested on animals or cultured rabbit and human cornea cells in vitro [17]. However, recently, regulations on animal testing and cultured animal and human cornea cells have been strengthened and testing further restricted. In this context, laboratory experiments on the rheological behavior of artificial tear solutions and modeling their dynamics can constitute alternatives to animal testing. Modeling can lead to a good understanding of the spreading mechanism of artificial tears in relation to their rheological properties. Furthermore, modeling can help laboratories design the most effective, longest-lasting, and most comfortable solutions on a rational basis and objective criteria.

Many models for the spreading of tear film can be found in the literature [18]. Most of these models are restricted to the aqueous layer, considered to be a Newtonian fluid, spreading under the actions of gravity, surface tension, viscosity, and evaporation during blinking [19,20,21]. These are single-layer models for the film thickness; they are solved in fixed and time-dependent domains (eye blinking) [22,23,24]. In the literature, there are also two-equation and three-equation models with coupled, non-linear partial differential equations for the thicknesses of the aqueous and non-polar lipid layers, and the concentration of the polar lipid layer at the interface between the aqueous and non-polar lipid layers [25,26]. The non-Newtonian behavior (shear thinning) of tear substitutes is modeled using the Ellis model. Jossic et al. discussed the spreading of shear-thinning tear substitutes during blinking [27]. They used a single-equation model for tear film thickness. They found that the shear-thinning propriety slows down the thinning of the tear film and delays its breakup. The authors also showed that the shear-thinning nature of the fluids improves the homogeneity and the stability of tears compared with a Newtonian-type substitute. However, it is known that Ellis’s model overestimates the effect of shear-thinning properties of tears [27]. Using the Cross model, Mehdaoui et al. investigated the shear-thinning properties of tears spreading over a spherical cornea [27].

Artificial tears are also based on replenishing or increasing the thickness of the tear film lipid layer (TFLL). The lipid layer reduces the surface tension and respreads the tear film during the post-blinking phase [28]. The typical thickness of the lipid layer is approximately 100 nm [29]; at this scale, the continuum mechanics formulation of fluid dynamics may fail to describe the fluid dynamics within the TFLL and comprehend its fundamental properties; instead, statistical mechanics (molecular dynamics simulations) should be used. The reader is invited to see the review that discusses molecular dynamics applied to the tear film lipid layer [30].

Gel-based artificial tears as a protection for the delicate cornea surface have been of interest for a long time [17]. Synthetic soft hydrogels, biocompatible and exhibiting rheological properties similar to a natural soft hydrogel, are designed in laboratories to alleviate dry eye syndrome [17,31,32]. These gels behave as elastic material under low shear stress and as viscous material when the stress is above a certain threshold [33]. Such yield-shear stress behavior can be described by the Herschel–Bulkley model [34]. The gel-based artificial formulations manifest sufficient viscosity to prolong ocular surface retention when the shear is low (eye fully open) and low viscosity to allow the spreading of the teardrop over the cornea during the blinking phase when shear is high [13,29]. In addition, prolonged adhesion of gel-based artificial tears to the ocular surface can stabilize the tear film, delay the appearance of dry spots, and improve comfort [13,34]. 

In this study, we use numerical simulation to investigate and evaluate the coating of the eye surface by tear gel described by the Herschel–Bulkley rheological model. We examine the influence of gel properties on its dynamics, including eye blinking. First, the rheological properties and the spreading of commercially available gel-based artificial tears are characterized. Then, the continuity and momentum equations are solved using the volume-of-fluid (VOF) method and the continuous-surface-force (CSF) model. Finally, the numerical model is validated using the results obtained for Newtonian tear film by Ayedmir et al. [24]. The outline of the paper is as follows. First, the results are presented and discussed in Section 2. Concluding remarks are presented in Section 3. Then, the physical problem, the set of governing equations, the prescribed boundary conditions, and the numerical method and its implementation are described, given, and discussed in Section 4.

## 2. Results and Discussion

**Figure 1:**
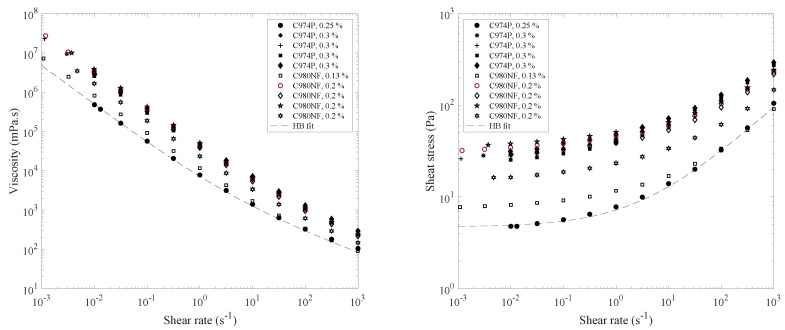
Flow curves of gel tears at 25 °C: yield stress and very high viscosity at a low shear rate and decreasing viscosity during a blink (high shear rate). The grey lines represent the Herschel–Bulkley model (Equation (1)) applied to the product with the lowest yield stress.

In this section, we present the results of our study of some commercially available carbomer gel-based artificial tears. Figure 1 shows the viscosity and the shear stress as a function of the shear rate. These flow curves emphasize that all carbomer-based products exhibit yield stress at a low shear rate and thin at a high shear rate. They also exhibit very high apparent viscosity (105 to 106 mPa·s) at low shear (10^−3^ s^−1^), and this viscosity is strongly reduced when the shear rate increases (about 10 mPa·s at 100 s^−1^). A percolated and disordered suspension of individual elastic sponges that absorb the solvent is a well-known characteristic of carbomer gels [35]. The rheological behavior of these products can be modeled with the Herschel–Bulkley (HB) constitutive model [36]:

(1) $$\{\begin{array}{c} \tau =\tau_{0}+k\begin{array}{cc} {\dot{\gamma }}^{n} & \end{array}\begin{array}{cc} \mathrm{if} & \begin{array}{cc} \tau >\tau_{0} & \end{array} \end{array}\\ \begin{array}{cc} \begin{array}{cccc} \begin{array}{cc} & \dot{\gamma }=0 \end{array} & & \mathrm{if} & \tau <\tau_{0} \end{array} & \end{array} \end{array}$$

*τ* being the stress (Pa), $\tau_{0}$ the yield stress (Pa), and k the consistency (Pa·s^n^). The dotted line in Figure 1 shows an example of the fitting of the HB model to the gel with the lowest yield stress. Table 1 summarizes the obtained values of the model parameters for all the gels. The shear-thinning index, n, differs very little between the products (0.4 to 0.5). The consistency, k, varies from 2 to 20 Pa·s^n^. The yield stress ranges from 4.7 to 33.8 Pa. More precisely, some products based on Carbopol^®^ 980 NF Polymer have a concentration of 0.2% and yield stress of about 30 Pa, whereas for the same concentration, other gels have a yield stress of about 15 Pa. The product with a lower concentration (0.13%) has lower yield stress (7.3 Pa). In the case of Carbopol^®^ 974 P Polymer, as the products have a concentration of 0.3%, their yield stress ranges from 26 to 28 Pa. The product with the lowest concentration (0.25%) has lower yield stress (4.7 Pa). The yield stress for each category of carbomer is not entirely correlated to its concentration. Indeed, the physicochemical characteristics and hence the formulation of the solvent may modify the behavior of carbomer microgels.

**Figure 2:**
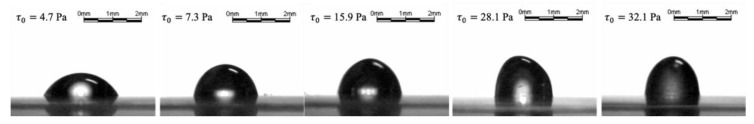
Spreading of gel tears with various yield stresses *τ*_0_.

Figure 2 shows the spreading over a PMMA substrate of several carbomer gels obtained with a drop of a similar size to those leaving bottles of eyedrops. Most drops of gel products do not spread to form a spherical cap on the PMMA. Instead, they form a heap that gradually slumps. The shape of the heap depends on the intensity of the yield stress. The tear gel with the lowest yield stress (4.7 Pa) forms a semispherical cap similar to Newtonian liquids. At the boundary between these two behaviors, the gel with a yield stress of 7.3 Pa spreads almost the same way as a Newtonian liquid. These results can be explained by a balance between yield stress and surface tension effects. The surface tension tends to minimize the interfacial energy by forming a semispherical cape, whereas the yield stress tends to stop the flow. We can introduce a plastic capillary number, $Ca_{P}=\tau_{0}R/\sigma$, where σ is the air/gel surface tension (= 65 mN/m), and R is the drop’s radius. The plastic capillary number is about 0.14 for the gel with a yield stress of 4.7 Pa and is larger than 0.5 for yield stress larger than 16 Pa. These results are essential for the modeling of the coating of the ocular surface by gel tears, as they show that if the yield stress is larger than about 7 Pa, they cannot spread over the surface during their instillation. Therefore, we limit ourselves to modeling gel products with yield stress less than 7 Pa.

### 2.1. Influence of the Tear Gel Rheological Parameters

This subsection explores the influence of the tear-gel rheological parameters, namely the behavior index, n, the consistency index, k, and the yield stress, *τ*_0_, on stability and tear film dynamics. Since tears present shear-thinning behavior, we considered values of n less than one. Although tear film breakup can occur at any position on the corneal surface because of TFLL structures (not considered here), the discussion below focuses on tear film breakup occurring just beneath the upper lid. Our results show that the lowest film thickness value is found near the upper eyelid, which is consistent with clinical observations [37]. The thinner the tear film, the greater its tendency to touch the corneal surface, and the higher the risk of tear breakup. In the following subsections, the graphs in the figures are flipped vertically, and 90° rotated compared with the scheme in Figure 9 in the section Materials and Methods. Hence, the moving upper eyelid is on the right side.

#### 2.1.1. Effect of Flow Behavior Index, n

**Figure 3:**
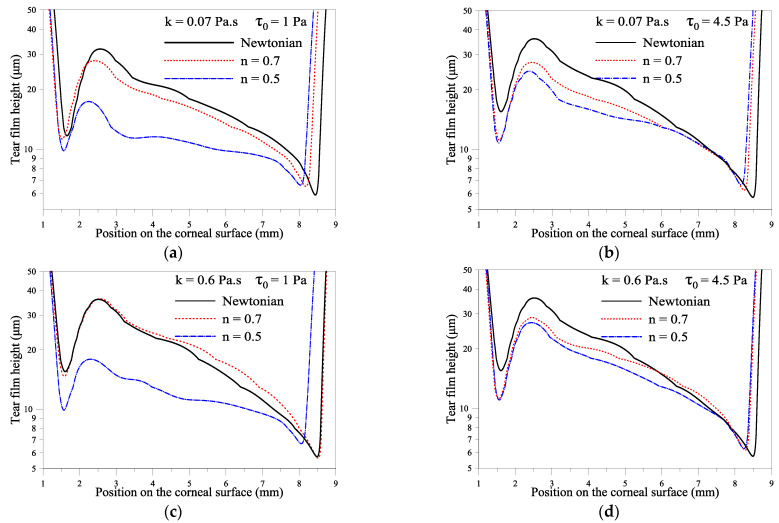
Effect of flow index, n, on variation of film thickness for *τ*_0_ = 1 and 4.5 Pa, k = 0.07 and 0.6 Pa·s at *t* = 0.18 s. *ρ* = 10^3^ kg·m^−1^, *μ* = 1.3 × 10^−3^ Pa.s (Newtonian case), *σ* = 0.045 N/m, h* = 0.0005 m, *L_cl_* = 0.001 m, *L_op_* = 0.01 m, *U_0_* = 0.0163 m/s. (**a**–**d**) indicate the same simulation with different values for the parameters indicated within the figure.

This subsection depicts the effect of the flow behavior index, n, parameter on the spreading of gel-based tears. We have considered two values for both the consistency and yield-stress parameters: k *=* 0.07 Pa·s and k *=* 0.6 Pa·s, *τ*_0_ =1 Pa and *τ*_0_ = 4.5 Pa, see Figure 3. Figure 3a–d indicates that the Newtonian tear films exhibit the lowest tear film thicknesses close to the upper eyelid than the gel-based tear films. Figure 3a–d suggests that gel-based tears can reduce the risk of tear film rupture near the moving upper eyelid. Figure 3a–d also indicates that the more the gel-based formulation exhibits a shear-thinning behavior (low n), the higher the minimum film thickness near the upper eyelid, and the more the risk of tear film breakup is reduced. The influence of shear-thinning is amplified by increasing the consistency parameter, k (see Figure 3a,c). Figure 3d shows that yield shear can compensate for the low shear-thinning effect (n = 0.7); see Figure 3c,d. It can be noted that when *τ*_0_ = 1 Pa and k = 0.07 Pa·s, the thickness profile tends to have a quasiuniform thickness over the center of the cornea flat. This result is interesting because any local changes in tear film thickness will result in an irregular air/tear interface, thus introducing aberrations to the eye’s optical system, which may cause the blurry vision commonly encountered in dry eye patients [38].

#### 2.1.2. Effect of Consistency Index, k 

**Figure 4:**
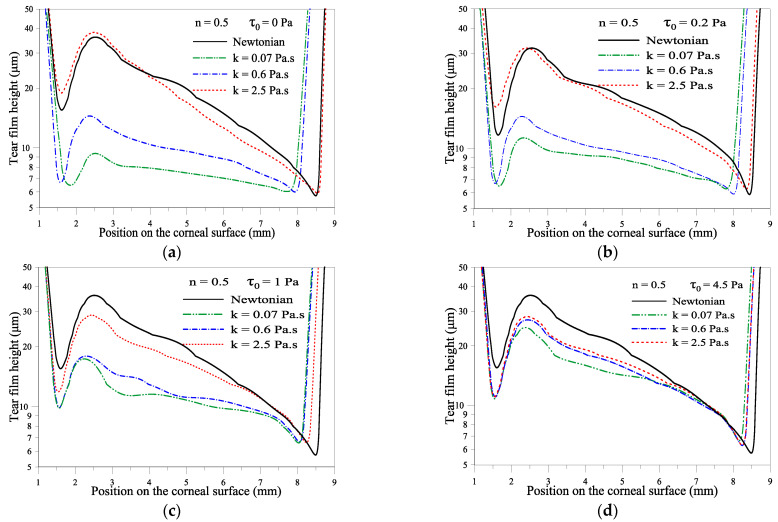
(**a**–**d**) the effect of consistency k, n= 0.5, *τ*_0_ = 0, 0.2, 1 and 4.5 Pa at *t* = 0.18 s.

Figure 4a–d depicts the influence of the consistency parameter k. In this figure, the value of the index parameter is fixed at n = 0.5. The yield stress *τ_0_* is fixed at 0, 0.2, 1, and 4.5 Pa. Figure 4a shows that the film thickness value decreases significantly near the lower fixed eyelid when the yield stress *τ*_0_ is null. The value of the consistency number is small, i.e., when the gel-based formulation does not exhibit elastic behavior (no yield stress) and the shear-thinning behavior of the gel formulation is not amplified. In this condition, the risk of tear film breakup is high near the lower eyelid. This thinning of the tear film near the fixed eyelid is alleviated by enhancing the elastic behavior (high value of *τ*_0_); see Figure 4d. The influence of the consistency parameter k on the film thickness near the upper moving eyelid is relatively small compared with the case of Newtonian tears when the values of the yield stress *τ*_0_ are null or low; see Figure 4a,b. The effect of the consistency parameter k is noticeable at higher values of the yield stress *τ_0_* because the elastic behavior is enhanced (high yield stress) and the shear-thinning behavior is amplified (high k); see Figure 4c,d. It is worth highlighting that the uniformity of the gel film thickness is better when the yield stress is null (Figure 4a). This means low values of yield stress help the gel to spread uniformly over the cornea.

#### 2.1.3. Effect of the Yield Stress *τ*_0_

**Figure 5:**
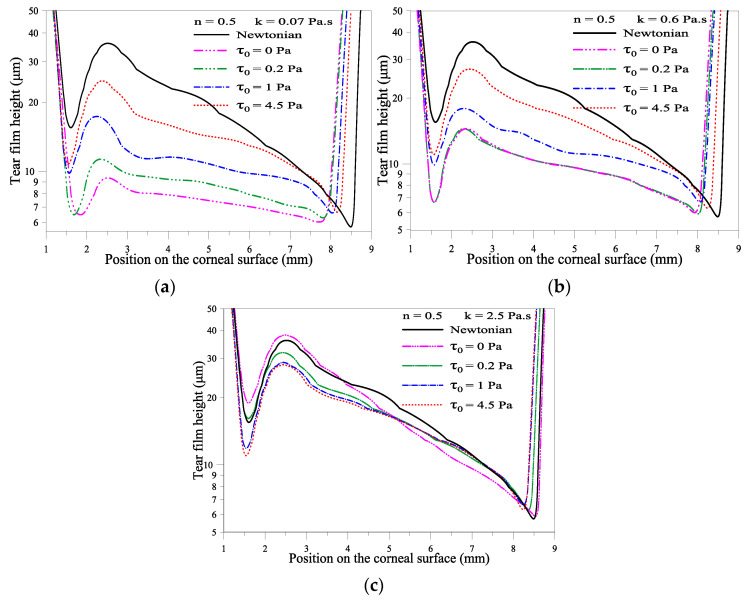
Variation of film thickness for different *τ*_0_, n = 0.5 at *t* = 0.18 s. (**a**) k = 0.07, (**b**) k =0.6, (**c**) k =2.5 Pa·s.

In this subsection, we discuss the influence of yield stress *τ_0_* on the tear film thickness profile. The flow index and the consistency index values are fixed at n = 0.5 and k = 0.07, 0.6, and 2.5 Pa·s. Figure 5a shows that for low consistency value (k = 0.07 Pa·s), increasing yield stress *τ_0_* increases the minimum film thickness near both eyelids. On the other hand, for consistency index *k* fixed at 2.5 Pa·s, we observe that *τ_0_* = 1 Pa is a cutoff value. Above this cutoff value, the minimum film thickness decreased significantly, which means that higher yield stress and the amplification of the shear rate contribution to the gel viscosity can break the tear film. When k = 2.5 Pa·s and *τ*_0_ = 4.5 Pa, the gel film is thinner in the lower part of the cornea and thicker in the upper part of the cornea. This non-uniform distribution of the gel film can blur the vision. Table 2 summarizes our results indicates that the following value of n = 0.5, k = 0.6 Pa·s, and *τ*_0_ = 1 Pa results in the highest value for the minimum gel-based tear film. One can iterate these values to design optimal gel-based artificial tears to alleviate the tear film breaking phenomenon.

#### 2.1.4. Evolution of Local Velocity at the Eyelids

**Figure 6:**
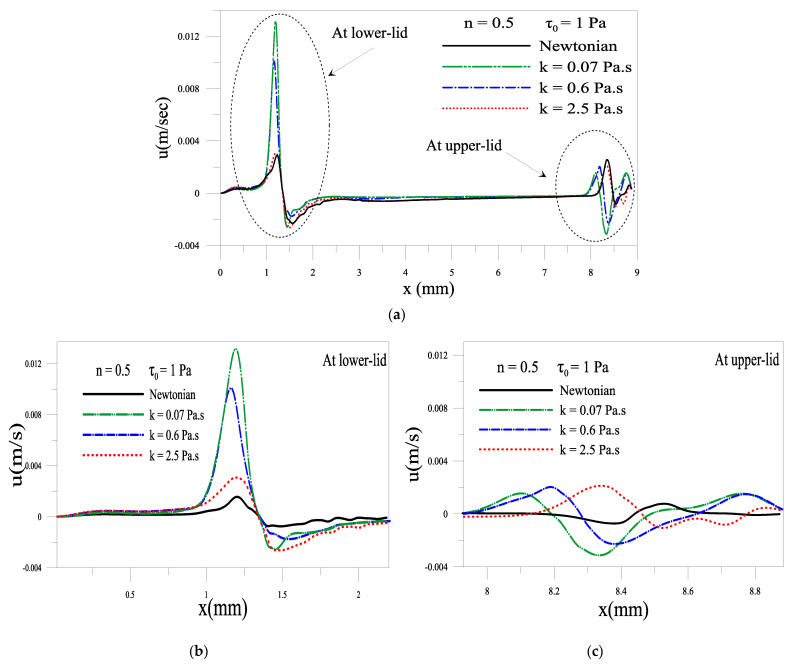
Depth average velocity for different consistency k, at time = 0.18 s. (**a**) over the whole cornea surface (**b**,**c**) a closeup near the lower and upper eyelid.

**Figure 7:**
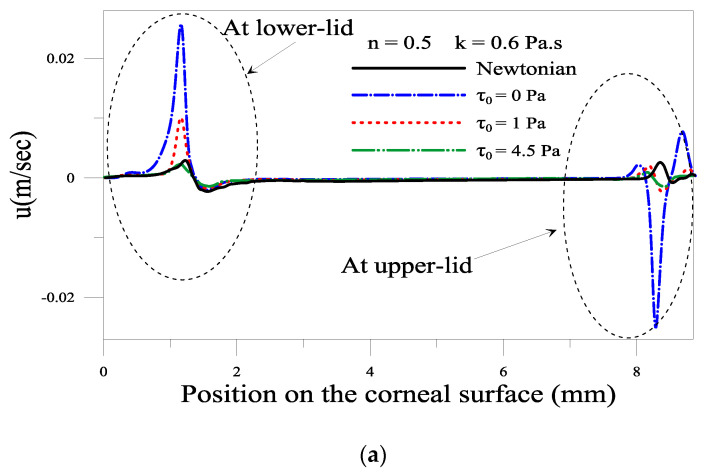

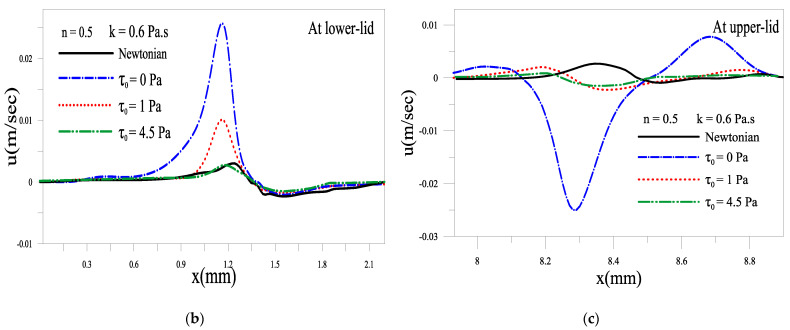
Depth average velocity for different. yield stress *τ_0_* at time = 0.18 s. (**a**) over the whole cornea surface, (**b**,**c**) a closeup near the lower and upper eyelid.

Figure 6 and Figure 7 depict the influence of consistency and yield stress on the average depth velocity of the gel-based tear film near the lower and upper eyelid. At the instant *t* = 0.18 s, the eye is fully open, and the upper eyelid is no longer in motion one can notice the existence of a backflow (negative velocity) near the lower and upper eyelid in the case of a gel-based tear. The backflow is more pronounced when the tear formulation presents no elastic behavior. However, there is almost no such backflow in the case of Newtonian tears. The backflow of gel film indicates that the gel climbs up the eye’s surface when the eyelid is at rest. Such backflow can prevent tear breakup near the eyelids. This backflow is significant when the values of consistency k and the yield stress *τ*_0_ are low; see Figure 6 and Figure 7a,b. This result mains a low gel viscosity eases its flow.

#### 2.1.5. Shear Stress of the Tear Film near Eyelids

**Figure 8:**
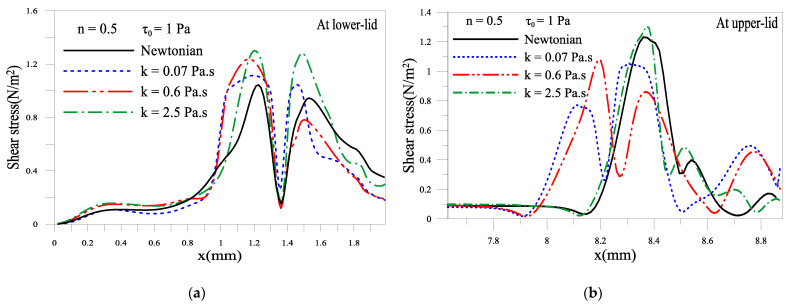

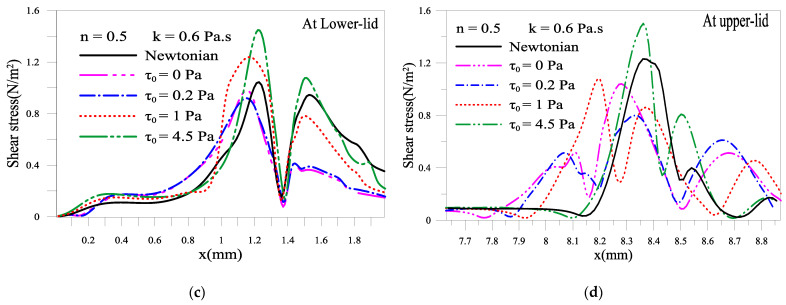
Variation of wall shear stress: (**a**,**b**) consistency *k* effect; (**c**,**d**) yield stress *τ*_0_ effect.

Figure 8 shows the effects of consistency and yield stress parameters on the shear stress near the eyelids. Shear stress at the cornea is an essential factor that one should consider when designing artificial tears. During the blinking phase, the shear forces transmitted to the cornea surfaces can damage the cornea’s cells and cause painful dragging sensations. Figure 8a,b shows that shear stress increases as the consistency index increases. This result is expected; the shear stress increases with the amplification of the shear rate effect. Figure 8c,d also shows that increasing yield stress augments the shear stress. This is also is an expected result.

## 3. Conclusions

In this paper, we have presented the results of our study carried out on some commercially available gel-tear substitutes based on carbomer. The experiment shows that the yield stress for each category of carbomer is not entirely correlated to its concentration. Indeed, the physicochemical characteristics and hence the formulation of the solvent may modify the behavior of carbomer microgels. Moreover, we have shown that the Herschel–Bulkley model can describe this type of gel-based artificial tear. Furthermore, we have demonstrated that gel with yield stress above 7 Pa·s does not spread over the surface, which led us to consider low yield stress gels in the present study. We have explored the dynamics of gel-based artificial tears using the Herschel–Bulkley model. The spreading dynamics of such artificial tears are explored on planar subtract, including blinking. We have investigated the influence of the three parameters of the Herschel–Bulkley model, namely yield stress *τ*_0_, flow behavior index n, and the consistency index k on the spreading gel-based tear film. We found that enhancing the shear-thinning of the gel-based tears by decreasing the flow behavior index *n* contributes to increasing the value of the minimum film thickness, which confirms the positive effect of the shear-thinning properties of the natural tears. Low value for the yield stress tends to delay the film breakup and ensures quasi-uniform film thickness around the center of the cornea, which can prevent blurred vision when using gel-based artificial tears. In the range of parameters considered in this research, the maximum value for the minimum tear film is found for values of the gel parameter n = 0.5, *τ*_0_ = 1 Pa and k = 0.6 Pa·s. These values can serve as a starting point for an iterative and optimization process to reach perhaps an optimal design of artificial gel-based tear film. We believe that a modeling approach similar to the one presented here can help laboratories design tears on rational basis to alleviate dry eye symptoms.

## 4. Materials and Methods

### 4.1. Rheology and Spreading of Tear Substitutes

The molecules used in tear substitutes are viscosifiers that modify the rheological behavior of an aqueous solution. We studied ten carbomer-based gel substitutes. Table 1 summarizes the viscosifiers used and their concentration for each product. The viscosity measurements in shear and steady regimes were carried out with a TA Instruments ARG2 high-sensitivity controlled torque rotational rheometer. A cone-plate measurement configuration was selected. The advantage of this geometry lies in the fact that it generates homogeneous and controlled shear flow throughout the entire sample. We recall that the dynamic viscosity of the material is defined by

(2) $$\mu =\frac{\tau }{\dot{\gamma }}$$

with μ being the dynamic viscosity (Pa·s), τ is the shear stress (Pa), and $\dot{\gamma }$ is the shear rate (s^−1^). The temperature of the sample was regulated by an integrated Peltier effect system that heats the plate of the cone-plate measurement configuration. A Pt 100 probe controls its temperature. The regulation system ensured that the temperature of the lower plate was accurate to within ±0.1 °C. The measurement geometry was enclosed in an envelope that acts as a solvent trap, thus considerably reducing evaporation on the unconfined surface of the sample. It also reduced heat losses and ensured a uniform temperature around the sample. A rough geometry is used to avoid wall slip (cone 26 mm in diameter, 4° angle, 450 μm gap) [39]. First, a sample was taken directly from its receptacle and placed on the rheometer plate. The cone was then brought up to the plate until the required gap is obtained. We gave enough time to balance the temperature at 25 °C, before applying a shear rate or stress, and the change in torque (stress) or strain is recorded as a function of time until steady conditions are achieved.

Gel spread measurements were performed using the Digidrop device (Contact Angle Meter, GBX, Valence, France). This device consists of a camera (25 images/s), a plate thermostated at 25 °C on which the substrate is placed, and a syringe holder that can translate vertically. A 1.37 mm inner diameter and 1.65 mm thick polypropylene needle allow drops of a size similar to those made by eye drops bottles, whose radius is less than the capillary length. The measurements were carried out by slowly depositing a drop of tear gel on a PMMA substrate. PMMA substrates were supplied by Goodfellow (Friedberg, Germany). The surface energy of PMMA (*γ*_SA_) is 40 mN/m at 20 °C. It is hydrophobic. The PMMA/water contact angles approach those epithelium/water. 

### 4.2. Numerical Simulations

The physical domain and its corresponding numerical model, delimited by a flat vertical substrate and two eyelids, are shown in Figure 8. The tear volume initially squeezed between closed eyelids spreads over the cornea surface to form a protective tear film with the motion of the upper eyelid while the lower eyelid remains fixed. The distances between the eyelids in open and closed positions are *L_op_* = 10 mm and *L_cl_* = 1mm, respectively. The meniscus is pinned to the eyelids during the simulations at the height of h* = 0.5 mm. *L(t)* indicates the distance between the eyelids during the opening phase. *U(t)* is the velocity function of the upper eyelid. The domain is considered two-dimensional.

#### 4.2.1. Formulation of the Problem

The present study considers the tear film as a single layer with a minimum thickness of about 1 µm. At this scale, the continuum mechanics formulation of the tear film flow can be used. Therefore, the two-dimensional tear film flow can be described by the following Cauchy equations and continuity equations from hydrodynamic theory.

##### Governing Equations

The mass and momentum (Cauchy) conservation equations for the phase mixture within the physical domain are:

(3) $$\frac{\partial \rho_{m}}{\partial t}+\frac{\partial \left(\rho_{m}u\right)}{\partial x}+\frac{\partial \left(\rho_{m}v\right)}{\partial y}=0$$

(4) $$\frac{\partial \left(\rho_{m}u\right)}{\partial t}+\frac{\partial \left(\rho_{m}uu\right)}{\partial x}+\frac{\partial \left(\rho_{m}uv\right)}{\partial y}=-\frac{\partial p}{\partial x}+\frac{\partial \tau_{xx}}{\partial x}+\frac{\partial \tau_{xy}}{\partial y}-\rho_{m}g_{x}+F$$

(5) $$\frac{\partial \left(\rho_{m}v\right)}{\partial t}+\frac{\partial \left(\rho_{m}uv\right)}{\partial x}+\frac{\partial \left(\rho_{m}vv\right)}{\partial y}=-\frac{\partial p}{\partial y}+\frac{\partial \tau_{xy}}{\partial x}+\frac{\partial \tau_{yy}}{\partial y}+F$$

(*u,v*) are the velocity components, *p*, *g,* and $\rho_{m}$ are the pressure, gravitational constant, and density of the phase mixture, respectively. *F* is the source term due to the surface tension, defined in (Equation (9)). 

The free surface of the liquid film is resolved using the volume of fluid method (VOF). The conservation equation describes the dynamics of the interface:

(6) $$\frac{\partial \alpha }{\partial t}+\frac{\partial \left(\alpha u\right)}{\partial x}+\frac{\partial \left(\alpha v\right)}{\partial y}=0$$

The VOF method is based on the volume fraction field *α* having the values as follows:

α = 0: the cell is empty.
α = 1: the cell is full.
0 < α < 1: the cell contains the interface between the two fluids.

The following constitutive relations describe the characteristics of the fluid for the phase mixtures:

(7) $$\rho_{m}=\alpha \rho_{l}+\left(1-\alpha \right)\rho_{g}$$

(8) $$\mu_{m}=\alpha \mu_{l}+\left(1-\alpha \right)\mu_{g}$$

The subscripts “l” and “g” denote the liquid and the gas phases, respectively. The values of the density and the dynamic viscosity in the case of Newtonian tears are: $\rho_{l}\;$= 1000 kg·m^–3^, $\rho_{g}\;$= 1.225 kg·m^–3^ and $\mu_{g}$ = 1.7894 × $10^{-5}$ Pa·s, $\mu_{l}$ = 1.3 × $10^{-3}$ Pa·s.

As indicated above, the effect of surface tension is included in the source term, *F*, in (Equations (4) and (5)). The continuous surface force model (CSF) developed by Brackbill et al. [40] is used to account for the surface tension at the film interface as follow:

(9) $$F=\frac{\rho_{m}.\sigma .\kappa .\Delta \alpha }{\frac{1}{2}(\rho_{l}+\rho_{g})}$$

where *σ* is the surface tension, considered constant and equal to σ = 0.045 N/m, and κ is the curvature of the film. The latter writes:

(10) $$\kappa =\nabla .n=\frac{1}{\left|n\right|}\left[\left(\frac{n}{\left|n\right|}.\nabla \right)\left|n\right|-\left(\nabla .n\right)\right]$$

Here n is the normal vector to the free surface or normal gradient of α.

The deviator part of the stress tensor is given by the constitutive law of Herschel–Bulkley fluid [36]:

(11) $$\{\begin{array}{c} \tau =\left(\frac{\tau_{0}}{\left|\dot{\gamma }\right|}+k{\left|\dot{\gamma }\right|}^{n-1}\right).\begin{array}{cc} \dot{\gamma } & \end{array}\begin{array}{cc} & \left|\dot{\gamma }\right|\neq 0 \end{array}\\ \begin{array}{cc} \begin{array}{cccc} \begin{array}{cc} \left|\tau \right|<\tau_{0} & \end{array} & & & \end{array} & \left|\dot{\gamma }\right|=0 \end{array} \end{array}$$

$\dot{\gamma \;}$ is the shear rate tensor and $\left|\dot{\gamma }\right|$ is its magnitude.

(12) $$\dot{\gamma }=\frac{1}{2}\left(\nabla u+\nabla u^{T}\right)$$

(13) $$\left|\dot{\gamma }\right|={\left(\dot{\gamma }:\dot{\gamma }\right)}^{1/2}$$

∇*u* is the velocity gradient and ∇*u*^*T*^ is its transpose. Thus, the viscosity of Herschel–Bulkley fluid is defined by the following relation [41].

(14) $${µ}_{l}=\frac{\tau_{0}}{\left|\dot{\gamma }\right|}+k{\left|\dot{\gamma }\right|}^{n-1}$$

The Herschel–Bulkley model has three parameters, assumed as constant, namely: *k* (the consistency index), $\tau_{0}$ (the yield stress), and n (the power index). $\left|\dot{\gamma }\right|$ is the shear rate. The yield stress *τ*_0_ plays the role of a discontinuous limit; Herschel–Bulkley fluid only flows when the shear stress exceeds the yield stress. $\tau <\tau_{0}$ the material remains rigid ($\left|\dot{\gamma }\right|=0\;$) and for $\tau >\tau_{0}$ the material flows as a power-law fluid ($\left|\dot{\gamma }\right|>0$). A regularization procedure is required to handle the discontinuity of the Herschel–Bulkley model at $\left|\dot{\gamma }\right|=0$. We used the regularization already implemented in the Fluent solver [42].

(15) $$\mu_{l}=\{\begin{array}{l} \frac{\tau_{0}}{\left|\dot{\gamma }\right|}+k{\left(\frac{\left|\dot{\gamma }\right|}{{\dot{\gamma }}_{c}}\right)}^{n-1}\begin{array}{cc} & \left|\dot{\gamma }\right|>{\dot{\gamma }}_{c} \end{array}\\ \frac{\tau_{0}}{{\dot{\gamma }}_{c}}\left(2-\frac{\left|\dot{\gamma }\right|}{{\dot{\gamma }}_{c}}\right)+k\left((2-n)+(n-1)\frac{\left|\dot{\gamma }\right|}{{\dot{\gamma }}_{c}}\right)\begin{array}{cc} & \left|\dot{\gamma }\right|<{\dot{\gamma }}_{c} \end{array} \end{array}$$

This regularization introduces an extra rheological parameter, the critical shear rate ${\dot{\gamma }}_{c}$ (which does not have a physical meaning) in the new definition of viscosity.

In the following, the value ${\dot{\gamma }}_{c}$ is fixed as ${\dot{\gamma }}_{c}=0.001\;s^{-1}$.

The consistency index k in the original Herschel–Bulkley model becomes equal to the ratio $\frac{k}{{\left({\dot{\gamma }}_{c}\right)}^{n-1}}$ in the regularized Herschel–Bulkley model in Fluent.

##### Boundary Conditions

The velocity vector *v* is subject to the Dirichlet boundary conditions, where the two components *u* and *v* correspond to the tangential and normal components on the corneal surface, respectively:

u=v=0                       y=0  : at the corneal surface.
u=v=0                       x=0  : at the lower eyelid.
u=U(t)                         x=L(t) :  at the upper eyelid.

L(t) is given in [24,43]:

(16) $$L(t)=\{\begin{array}{l} L_{cl}+U_{0}T\left(-\frac{1}{2}{\left(\frac{t}{T}\right)}^{2}+\lambda \left[\frac{\sqrt{\pi }}{2}erf\left(\sqrt{\frac{t}{T}}\right)-\sqrt{\frac{t}{T}}-e^{-t/\tau }\right]\right)\begin{array}{cc} & 0\leq t\leq t^{*} \end{array}\\ L_{op}\begin{array}{cc} & t>t^{*} \end{array} \end{array}$$

The velocity function *U(t)* of the upper eyelid was obtained experimentally after curve fitting the data by Wong et al. [19].

(17) $$U(t)=\{\begin{array}{c} U_{0}\left(\lambda \sqrt{\frac{t}{T}}e^{-t/T}-\frac{t}{T}\right)\begin{array}{cc} & 0\leq t\leq t^{*} \end{array}\\ 0\begin{array}{cc} & t>t^{*} \end{array} \end{array}\;$$

The approximate values for the parameters t*, *U*_*0*_, *T*, and *λ* are 0.180 s, 0.0163 m/s, 0.0865 s, and 11.6, respectively, for more details (see [43]). t* indicates the time at which the upper eyelid velocity becomes null. 

#### 4.2.2. Numerical Method and Validation

The numerical simulations were conducted using CFD commercial software (Ansys-FLUENT-17.0). The volume of fluid (VOF) method and the Continuous Surface Force (CSF) model are adopted in our case. We used the implicit scheme discretization to solve the governing equations. The calculation was continued until the eye became fully open, t = 0.18 s. In this work, convective terms of Navier–Stokes equations are discretized with the second-order upwind scheme. Speed-pressure coupling is treated with SIMPLE algorithm, and pressure interpolation was done using the PRESTO scheme. FLUENT uses a first-order non-iterative scheme with a variable time step to integrate the transient term of the filling rate equation (Equation (6)). The time step simulation has been kept at 2 × 10^−6^. The convergence criterion is chosen so that the residual for all equations is less than 10^−7^.

**Figure 9:**
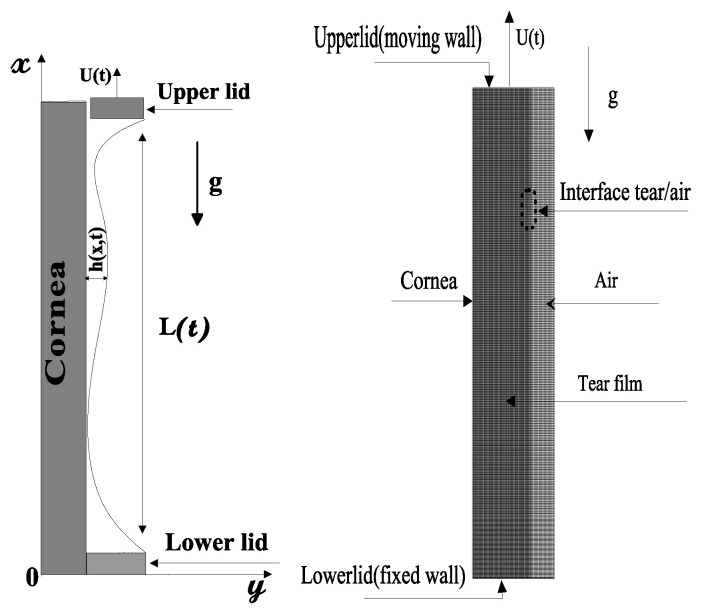
Physical and numerical domain of the tear film problem.

**Figure 10:**
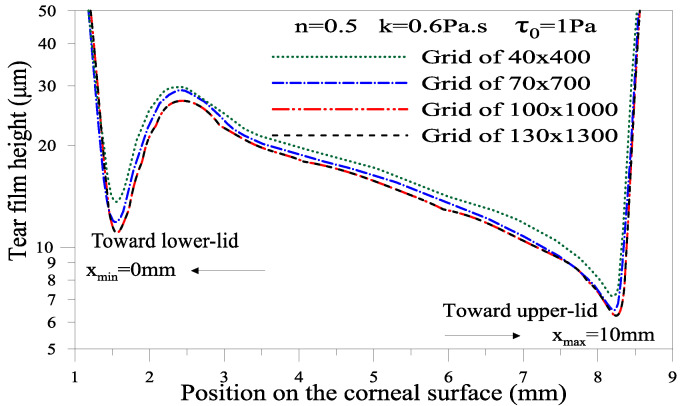
Evolution of film thickness for different mesh grids in the case of non-Newtonian film *n* = 0.5, *k* = 0.6 Pa·s and *τ*_0_ = 1 Pa at *t* = 0.18 s.

A dynamic uniform mesh grid and the refinement near the cornea are used to account for the moving upper eyelid. The upper eyelid velocity (Equation (17)) is implemented as a user-defined function (UDF). Mesh sensitivity was performed using different grids from 40 × 400 to 130 × 1300. The calculations show that the film thickness profile becomes insensitive to a mesh grid of 100 × 1000 when a gel tear film is considered; see below. Compared with the scheme in Figure 9, the graphs in Figure 10 and the figures above (Results and Discussion section) were flipped vertically and rotated 90°. Hence, the moving upper eyelid is on the right side. The deviations in the thickness of the tear film calculated using a 130 × 1300 grid are almost null compared with those computed using a 100 × 1000 grid. Therefore, the rest of our computations were performed with a 100 × 1000 grid for both Newtonian and non-Newtonian tears.

**Figure 11:**
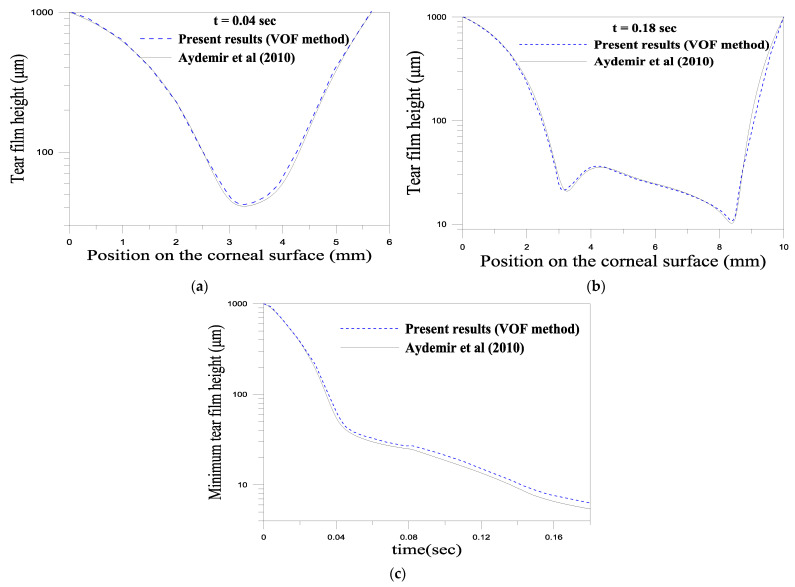
Model validation against the model by Aydemir et al.’s work [24]. (**a**,**b**) film thickness distribution along the planar subtract at 0.04 and 0.18 sec, respectively. (**c**) Evolution of the minimum tear film thickness with time. The parameters of the simulations are: *ρ* = 10 ^3^ kg·m^−1^, *μ* = 1.3 × 10^−3^ Pa.s, *σ* = 0.045 N/m, h* = 0.001 m, *L_cl_* = 0.002 m, *L_op_* = 0.01 m, *U_0_* = 0.0163 m/s.

The computational results have been validated using the results of the lubrication theory by Aydemir et al. [24]. This validation is illustrated in Figure 11. Figure 11a,b shows good agreement between the numerical and lubrication models during the opening phase. However, one can notice a slight difference in the minimum thickness (see Figure 11c). Therefore, we believe that the results obtained by solving the full governing equation should be more accurate than those obtained by the reduced model.

## Figures and Tables

**Table 1:** Results for gel tear substitutes at 25 °C.

Composition	Concentration (%)	Yield Stress (Pa)	k (Pa.s^n^)	n
Carbopol^®^ 974 P	0.25	4.7	2.5	0.5
	0.3	23.8	18.1	0.4
	0.3	26	14.4	0.4
	0.3	27.7	20.5	0.4
	0.3	28.1	16.9	0.4
Carbopol^®^ 980 NF	0.13	7.3	3.4	0.5
	0.2	15.9	7.9	0.4
	0.2	31.3	12.4	0.4
	0.2	32.1	15	0.4
	0.2	33.8	15.5	0.4

**Table 2:** Minimum film thickness of viscoplastic tear at the end of blinking (t = 0.18 s).

		h_min_ at Upper-Lid (10^−7^ m)			
		*τ*_0_ = 0 Pa	*τ*_0_ = 0.2 Pa	*τ*_0_ = 1 Pa	*τ*_0_ = 4.5 Pa
n = 0.5	Newtonian	0.574			
	k = 0.07 Pa·s	0.603	0.628	0.660	0.664
	k = 0.6 Pa·s	0.599	0.591	0.671	0.625
	k = 2.5 Pa·s	0.589	0.627	0.641	0.631
